# Effects of Introducing a Secure Web-based Messenger Application for Communication Among Non-consultant Hospital Doctors (NCHDs)

**DOI:** 10.7759/cureus.5285

**Published:** 2019-07-31

**Authors:** Mel Corbett, Lucy Chapman, John O'Shea, Patrick Canning, Salim Sebaoui, Donnchadh O'Sullivan, Norah Power, Margaret O'Connor

**Affiliations:** 1 Otolaryngology, University Hospital Galway, Galway, IRL; 2 Ageing and Therapeutics Division, University Hospital Limerick, Limerick, IRL; 3 Computer Sciences and Information Systems, University of Limerick, Limerick, IRL; 4 Paediatrics, University Hospital Limerick, Limerick, IRL

**Keywords:** whatsapp, safety, communication, data protection, information governance, mobile, non-consultant hospital doctors (nchds)

## Abstract

Introduction: Use of web-based messaging applications to communicate clinical information is common between non-consultant hospital doctors (NCHDs). This study sought to assess web-based messenger use in NCHDs following the introduction of a more secure alternative to WhatsApp (WhatsApp, Inc., Menlo Park, CA, USA).

Methods: A 10-item survey was undertaken on two NCHD cohorts. The second cohort received training on data protection and an alternative application to WhatsApp. Quantitative data analysis was conducted using the IBM Statistical Package for Social Sciences (SPSS) (IBM SPSS Statistics, Armonk, NY).

Results: The total response rate across both groups was 63% (N = 68). The majority of respondents used WhatsApp to communicate clinical information. In the second cohort, fewer NCHDs shared identifiable sensitive patient information 97% (n = 29/30) vs 81% (n = 25/31) and fewer NCHDs shared/stored clinical images.

Discussion: WhatsApp use is common among NCHDs. An alternative means of communication can improve the safety of patient data. NCHDs require more training on data protection laws and their own responsibilities.

## Introduction

Modern-day healthcare involves large volumes of sensitive information to be communicated rapidly and securely on a continuous basis. With increasing amounts of sensitive patient information comes an increasing requirement for this information to be processed and communicated between hospital staff. Miscommunication between hospital staff is a major factor in adverse events [1]. In order to prevent errors occurring due to communication deficits, there has been an increasing focus on training of personal communication skills [2]. Communication improvements between the physician and patient can result in greater patient satisfaction and fewer claims of malpractice [3]. Specifically, literature surrounding communications between doctors have shown an increasing need for training in order to reduce miscommunication and errors [4].

Surgical specialties now incorporate human factors training into all programmes in an effort to improve communications among non-consultant hospital doctors (NCHDs) [5]. Efficient communication facilitates good patient care. With current paper or electronic records, occasionally physicians require more direct communication to clarify management plans, communicate results, or delegate work. Requirements are amplified at shift changeovers where miscommunication can result in a data breach or patient harm [6].

Hospital communication infrastructure relies on pager-based systems allowing doctors to communicate with colleagues via phone calls on receiving bleeps. Drawbacks of these traditional pager-based systems have been highlighted in the literature, including delayed communication in an emergency situation, inefficiency in terms of physician and nursing time, and the asynchronous one-way transmission of information [7]. Physicians can spend up to 20% of their allocated working time answering paging requests.

The Health Service Executive (HSE) offers little clear guidance or clarification on the appropriate use of web-based messenger apps for communication in a clinical setting. Current policies on social media usage and electronic communication do not offer guidance to staff communicating via messaging applications [8-9]. Minimal training is provided to NCHDs on patient data protection laws or appropriate conduct using these types of applications. Studies demonstrating the widespread use of WhatsApp (WhatsApp, Inc., Menlo Park, CA, USA) [10] are reflective of NCHDs in Ireland and the United Kingdom who are faced with the problem of needing to rapidly communicate large volumes of information to colleagues on a regular basis with restrictive hospital guidance on available strategies. The National Health Service (NHS) has recently changed its policies regarding the use of web-based messaging applications to provide clinicians with guidance on correct usage in the clinical setting [11]. These guidelines give clinicians advice on optimal usage practices and offer information on available third-party applications, recognising the requirement for mobile messaging in a clinical setting.

Ownership of this data sent through smartphones is not transparent, and there are issues surrounding the consent process for data use [12]. Sending data via applications, such as WhatsApp, can result in serious data breaches. The risk that people could be inappropriately added to groups containing sensitive patient information may be high, especially without a clear authorization process on accessing the application.

There is an evident need to separate work and social domains for NCHDs and placing work information in the same application as social information can contribute to miscommunication among NCHDs. The differing requirements of a hospital-appropriate, web-based messaging tool necessitate an alternative to the currently available applications for appropriate use of patient information.

## Materials and methods

This study sought to assess behaviour regarding sensitive patient information among NCHDs and to assess the effects of a hospital-approved application on these behaviours. A cross-sectional study of two groups of intern-level NCHDs was conducted in May 2018 and August 2018. NCHDs were surveyed over a one-week period and surveys were returned within two weeks. They were approached in person and given a copy of the survey by a gatekeeper who had no involvement with the study. Written informed consent was obtained from each participant. Consent forms were retained separate to the surveys and each survey was pseudonymised with a unique identifier. Group 1 was a cohort of interns had received no formal training on data protection responsibilities and had not been introduced to any approved application for communication of patient information.

Group 2 was a similar cohort of interns. These were given a brief talk on the importance of information governance and how new data protection legislation could affect them. The intern network coordinator, with the intention of preventing data breaches, gave this talk. The hospital had introduced Medxnote (Medxnote Ltd., Dublin, Ireland) as a secure web-based messaging application intended for communication of de-identified patient information. Teaching sessions were provided to ensure the NCHDs were capable of using the application. Each teaching session lasted approximately 30 minutes in duration and was provided by the application designer. One teaching session was deemed to be sufficient for most people using the app. The second group was surveyed following two months of working with access to the novel application and training. Technical support was offered during daytime hours in the event of difficulties in using the app.

The survey was contained 10 questions, which assessed the behaviour among NCHDs regarding sensitive patient information. These were analyzed using descriptive statistics in Microsoft® Excel (Microsoft® Corp., Redmond, WA) and comparison between groups made using the IBM Statistical Package for Social Sciences (SPSS) (IBM SPSS Statistics, Armonk, NY).

## Results

Among the first cohort of interns prior to the General Data Protection Regulation (GDPR) and prior to formal training in data protection responsibilities or any hospital-approved web messenger, the survey response rate was 33/54 (61%). Three surveys were filled out incorrectly and so only 30 were analyzed.

All respondents (n = 30) had smartphones. Password protection on smartphones was reported by the vast majority of respondents (97%, n = 29). Notably, all respondents (100%, n = 30) indicated that WhatsApp was the primary method of communication amongst their hospital team.

Twenty-seven respondents (90%) disclosed that they sent or received clinical information, including patient identifiers, on a daily basis via WhatsApp. Similarly, 90% (n = 27) sent or received pictures relating to patients or patient information via WhatsApp on a daily basis. Storage of pictures relating to clinical details or patient information on their mobile phones was acknowledged by 90% of respondents (n = 27). Over half (59%, n = 16) of these pictures containing clinical details and/or patient information were stored within WhatsApp, whilst 41% (n = 11) were stored in the respondents' personal picture gallery. Fifty percent of respondents regularly received clinical details of patients who were not under their team's care. Furthermore, 10% (n = 3) had accidentally sent patient information to the wrong contact or group of contacts in the prior month. Of these accidental transmissions of patient information, recipients were reported to be hospital staff.

**Figure 1:**
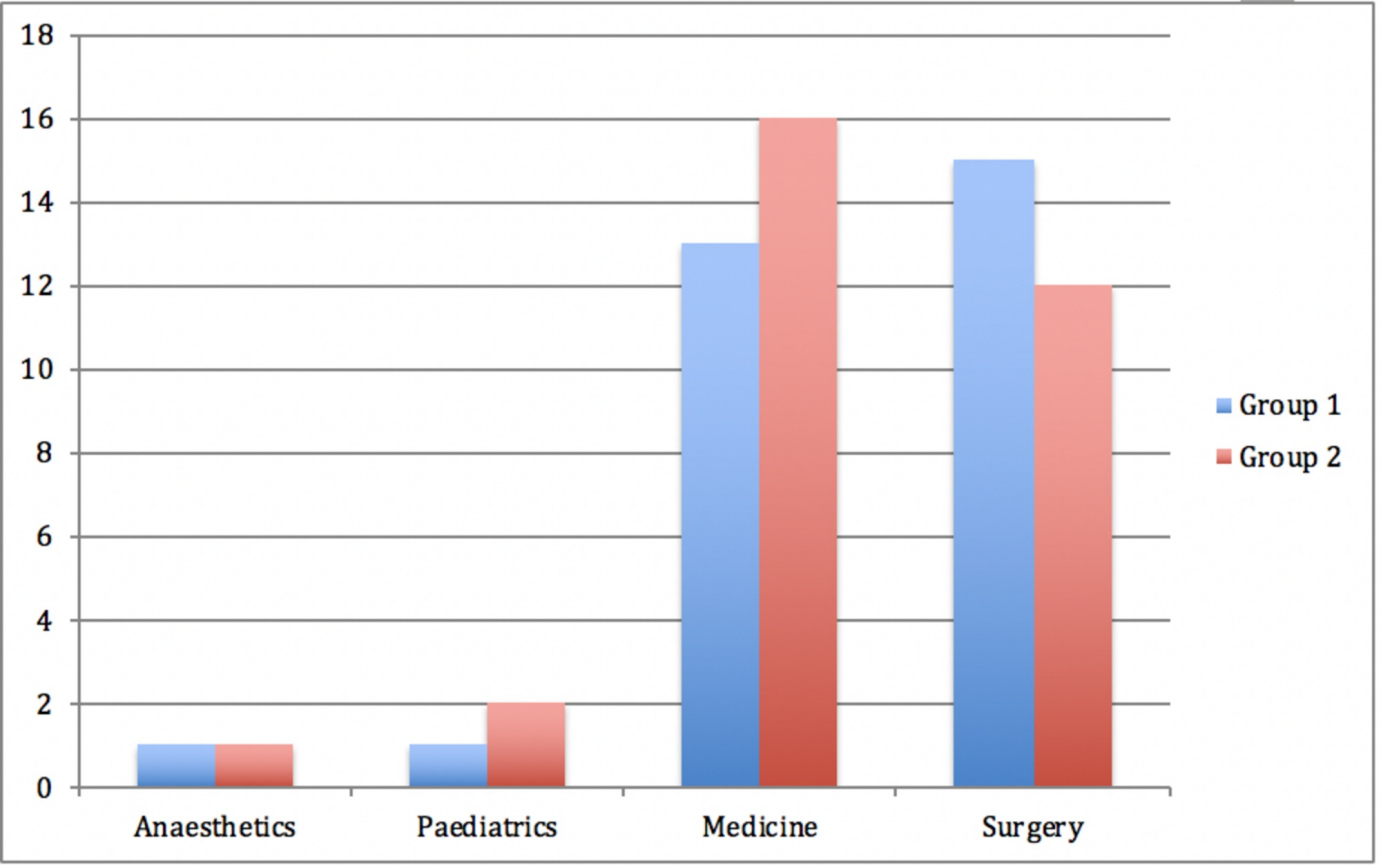
Departments of respondents

The second cohort response rate was 35/54 (65%) with four surveys answered incorrectly and deemed unsuitable for analysis, leaving 31/54 (57%) available for data analysis. The second group were all required to download the approved application to receive communications relating to announcements about teaching, training, and management. Figure 1 compares the specialties of each group of respondents.

Of the second group surveyed, 13/31 (42%) had attended the available teaching relating to the use of the approved application and GDPR.

Similar to the first cohort, all respondents (n = 31) surveyed had smartphones. The majority of respondents (94%, n = 29) had password protection on their phones. When asked about messaging application use, 19 respondents (61%) said that they sent or received clinical information, including patient identifiers, on a daily basis, whilst 19% (n = 6) reported this occurring on a weekly basis and 13% (n = 4) on a monthly basis. A total of five respondents (16%) reported neither sending nor receiving clinical information to colleagues via a messaging application.

**Figure 2:**
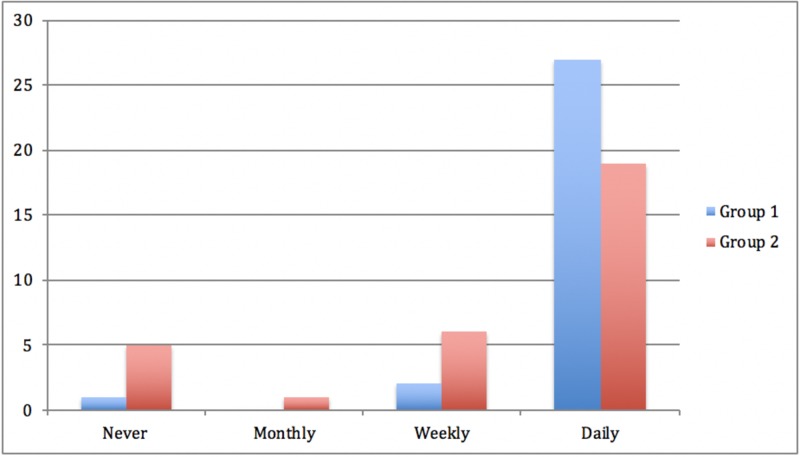
How often respondents sent or received identifiable sensitive patient information on their mobile phones

**Figure 3:**
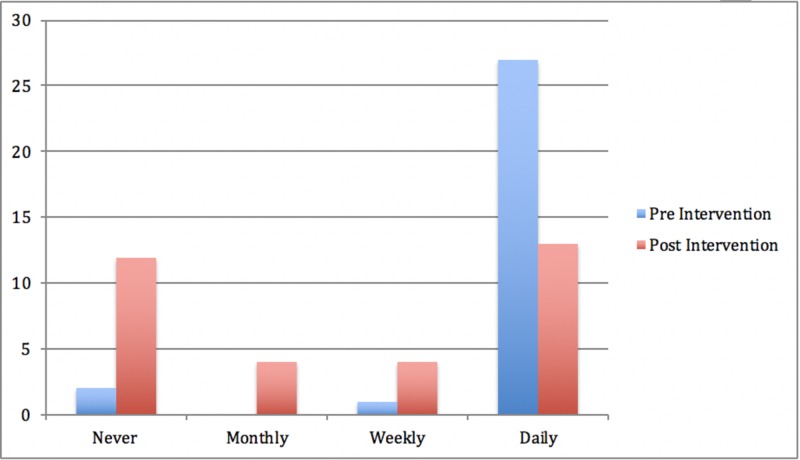
How often respondents sent or received identifiable clinical images on their mobile phones

Almost half of the respondents (42%, n = 13) sent or received pictures relating to patients or patient information via WhatsApp on a daily basis. Thirteen percent of respondents sent or received images on a weekly basis, and similarly, a further 13% reported this occurring on a monthly basis. Twelve respondents (39%) stored pictures relating to clinical details or patient information on their mobile phones. Of the subgroup who saved clinical images, 33% (n = 4) stored these pictures within the WhatsApp application and 66% (n = 8) stored clinical pictures in their smartphone picture gallery. One respondent (3%) admitted to accidentally sending clinical information to the wrong contact in the prior month. Figures 2-3 highlight the reduction in sharing sensitive patient information and sharing clinical images between groups

Statistical analysis was conducted using the SPSS software with the Chi-squared test employed to compare groups with a statistical significance  threshold set at p < .05. Table 1 compares the results of the survey questionnaire between both groups.

**Table 1:** Comparison of Groups

Question	Group 1 (N = 30)	Group 2 (N = 31)	P-value
Number who had no password protection on their phones	1 (0.33%)	2 (0.6%)	0.5734
Number who’s hospital teams used Whatsapp as the primary method of communication	30 (100%)	25 (81%)	0.024
Number who sent or received any identifiable patient information via the messaging application	29 (96.6 %)	25 (81%)	0.0496
Number who sent or received any identifiable clinical images via messaging application	28 (93.3%)	17 (55%)	< 0.001
Number who sent or received sensitive information more than once/month	29 (96.6%)	25 (81%)	0.0496
Number who sent or received identifiable clinical images more than once/month	28 (93.3%)	15 (48%)	< 0.001
Number who stored clinical images on their mobile phone	28 (93.3%)	12 (39%)	< 0.001
Number who accidentally sent information to the wrong contact	3 (10%)	1 (3%)	0.28
Number who accidentally sent information to non-hospital staff	0	0	1

## Discussion

With the introduction of GDPR, clinicians must communicate fast and accurate patient information while avoiding irresponsible usage of sensitive patient information. Following the training and the introduction of an application suitable for sending de-identified patient information, fewer respondents sent or received sensitive patient information and fewer respondents sent or received identifiable clinical images. Clinical images were saved to mobile phones less frequently.

These differences may be due to the new training and teaching available to the NCHDs. The presence of an application which is exclusively for professional communication in this group improved behaviours surrounding the use of patient information. Differences could also have been influenced by increasing awareness of the General Data Protection Regulation. Increased emphasis on appropriate usage of applications by hospitals reminds clinicians to maintain high standards in using sensitive patient information.

Further advances in mobile communication technology, along with national policies, will determine the future of web-based communications in hospitals. Broader research questions could examine the multitude of uses of web-based messenger applications and the different features of these applications. Factors which could have impacted on participants’ behaviour include training, the availability of the app, and the introduction of the GDPR.

Governing bodies at a local and national level can help improve information governance among NCHDs. Clinicians often come up with their own solutions to communicate clinical information, which can result in data breaches. Policies can recognise that usage of web-based messaging systems is sometimes necessary in the clinical setting and this usage can be regulated. Alternatives to pager-based systems can make hospital communication more efficient and can be used in a safe manner that is compliant with GDPR.

## Conclusions

Our results show that third-party web-based messenger applications are in widespread use in the clinical setting. Providing NCHDs with training on GDPR and an approved web-based messenger application has resulted in an improvement in the behaviour surrounding the sharing of sensitive patient information. Our study demonstrated a significant improvement in compliance with new standards of data protection following training and standardization of communication modes. Training and the appropriate software can help hospitals to comply with regulations and protect patients' data. Junior doctors are now more cognizant of their data protection responsibilities and the issues with current practices.
